# Accurate Post-Calibration Predictions for Noninvasive Glucose Measurements in People Using Confocal Raman Spectroscopy

**DOI:** 10.1021/acssensors.2c02756

**Published:** 2023-03-06

**Authors:** Anders Pors, Kaspar G. Rasmussen, Rune Inglev, Nina Jendrike, Amalie Philipps, Ajenthen G. Ranjan, Vibe Vestergaard, Jan E. Henriksen, Kirsten Nørgaard, Guido Freckmann, Karl D. Hepp, Michael C. Gerstenberg, Anders Weber

**Affiliations:** † RSP Systems, Sivlandvænget 27C, 5260 Odense, Denmark; ‡ Institute for Diabetes Technology at University of Ulm, Lise-Meitner-Straße 8/2, 89081 Ulm, Germany; § Steno Diabetes Center Copenhagen, Borgmester Ib Juuls Vej 83, 2730 Herlev, Denmark; ∥ Steno Diabetes Center Odense, Kløvervænget 10, 5000 Odense, Denmark; ⊥ University of Munich (emeritus), Geschwister-Scholl-Platz 1, 80539 Munich, Germany

**Keywords:** non-invasive glucose monitoring, in vivo Raman spectroscopy, portable sensor, calibration stability, multivariate data analysis, tissue diagnostics, diabetes

## Abstract

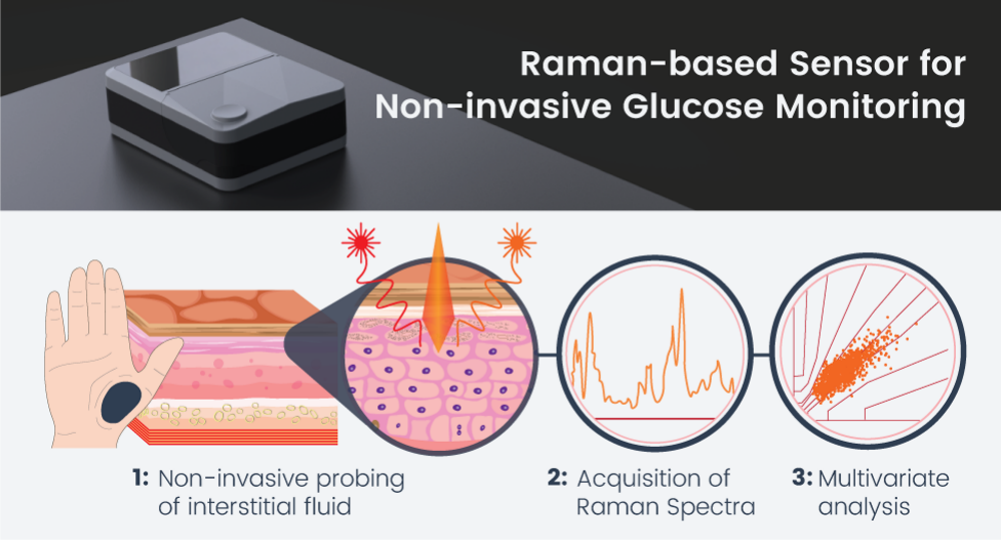

In diabetes prevention and care, invasiveness of glucose measurement impedes efficient therapy and hampers the identification of people at risk. Lack of calibration stability in non-invasive technology has confined the field to short-term proof of principle. Addressing this challenge, we demonstrate the first practical use of a Raman-based and portable non-invasive glucose monitoring device used for at least 15 days following calibration. In a home-based clinical study involving 160 subjects with diabetes, the largest of its kind to our knowledge, we find that the measurement accuracy is insensitive to age, sex, and skin color. A subset of subjects with type 2 diabetes highlights promising real-life results with 99.8% of measurements within A + B zones in the consensus error grid and a mean absolute relative difference of 14.3%. By overcoming the problem of calibration stability, we remove the lingering uncertainty about the practical use of non-invasive glucose monitoring, boding a new, non-invasive era in diabetes monitoring.

In diabetes prevention and care, invasiveness of glucose measurement impedes efficient therapy and hampers the identification of people at risk. Among non-invasive technologies such as electrical, thermal, acoustical, and optical methodologies, light offers the least intrusive probing of all technologies investigated. Raman spectroscopy in the near infrared has shown a consistent path of improvement, driven by advances in lasers, optics, detectors, and algorithms. Furthermore, direct manifestation of physiological glucose in Raman spectra has been demonstrated, testifying that Raman spectroscopy measures glucose in skin at physiological concentrations.^1,2^ Despite these encouraging trends, a clinically useful embodiment of this method has not yet materialized.^3,4^

Accuracy, calibration stability, and general robustness have been persistent challenges for non-invasive glucose monitoring.^3−5^ Chemometrics and machine learning algorithms are generally used to build multivariate regression models that are subsequently used to predict the glucose concentration. Most attempts, irrespective of the underlying technology, involve brief study periods, typically not more than a few hours, under controlled conditions, and the demarcation between calibration and validation has often not been distinct.^6−8^ It has not been demonstrated whether these encouraging in-clinic results, acquired under controlled and supervised conditions, can be generalized to real-life conditions, extended measurement periods, and usage by lay person.

We have successfully bridged the gap from technical proof of principle to a safe and reliable device, which can be operated by non-specialists at home. Previously, we reported our first successful development of a Raman spectroscopic prototype in persons with diabetes and described a critical depth for the confocal glucose determination in human skin^9^ and the performance during glucose challenge.^10^ In the latter study, the prototype demonstrated glucose kinetics akin to invasive continuous glucose monitors,^11^ thus suggesting glucose measurements in the interstitial compartment. The glucose in the interstitial fluid arrives primary through diffusion from the capillaries and, thus, represents a time-delayed version of the blood counterpart.^12^ The use of the interstitial compartment may influence the measurement accuracy, but it is generally not considered a significant obstacle for practical glucose monitoring.^13^

The purpose of this paper is to present the key elements of the prediction algorithm development, the clinical evidence of the performance and calibration stability, and the utility of this serially manufactured Raman non-invasive glucose monitoring (NIGM) device in subjects with type 1 and type 2 diabetes on insulin therapy.

## Results

### Design of the Non-Invasive Glucose Sensor

**Figure 1:**
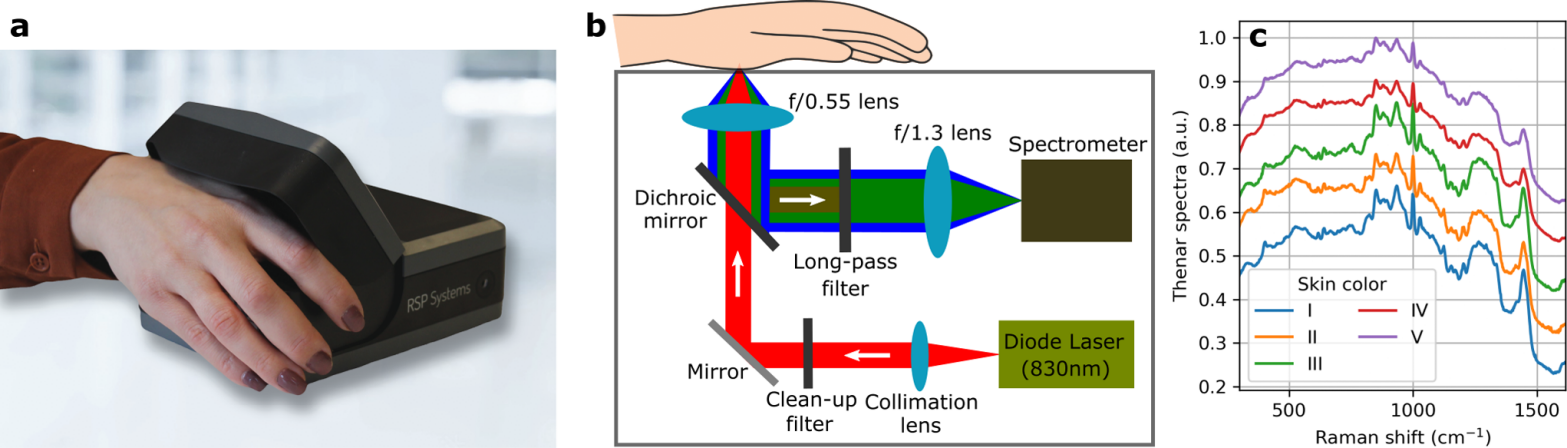
Non-invasive glucose sensor. (a) Novel, production-ready, portable, stand-alone, and Raman-based device configured for NIGM. (b) Schematic optical layout. (c) Examples of recorded thenar spectra from five subjects with different skin colors, according to the Fitzpatrick scale, where type I and type V correspond to the lightest and the darkest skin complexions, respectively. Spectra are vertically offset for clarity.

The sensor for non-invasive glucose determination is depicted in Figure 1a, where the hand is positioned as intended during use. The top cover functions both as a mechanical safety feature (for laser light irradiation) and screening of external light during measurements. It is worth noting that the sensor is portable, battery-driven, and with built-in safety measures, graphical user interface, and Wi-Fi connectivity. Moreover, the sensor is safe in use, which is corroborated by the fact that no serious reactions or scarring of the skin of the thenar (base of the thumb) was observed during the extensive clinical study.

The sensor’s optical components are presented schematically in Figure 1b and described in detail in the Materials and Methods section. Essentially, the optical hardware functions as a confocal near-infrared Raman spectrometer that is configured for maximum spatial sensitivity at 280 μm away from the glass window with a sensitivity profile featuring a full-width-at-half-maximum of 250 μm. With the thenar positioned on the window (Figure 1a), the confocal setup ensures that the backscattered Raman signal, arising from the interaction of the 830 nm laser illumination and the skin constituents, originates from the upper living skin layers (i.e., living epidermis and upper part of the dermis), while the signal from the dead outer skin layer (stratum corneum) is suppressed.^9^ Additionally, the confocality is helpful in reducing the dependency of the device-skin interface on the collected Raman signal, which amounts in more consistent Raman spectra.

The backscattered Raman signal is collected and dispersed by a spectrometer in the range of 300–1615 cm^–1^ with a spectral resolution of ∼10 cm^–1^. Figure 1c shows examples of recorded thenar Raman spectra from subjects with different skin colors (measured on a Fitzpatrick scale). It is comforting to see that despite slightly less pronounced Raman peaks for the darker skin colors (type IV and V), owing to an increased fluorescence background, the thenar spectra do not markedly differ, thus illustrating a relatively consistent response from all subjects. It is important to realize that the information of the physiological glucose concentrations resides in the thenar spectra, which can be quantified by the use of multivariate regression techniques. Details of the employed predictive algorithm, including pre-processing of spectra, detection of outliers, and training of calibration models, can be found in the Materials and Methods section.

### Maintenance of Calibration

**Figure 2:**
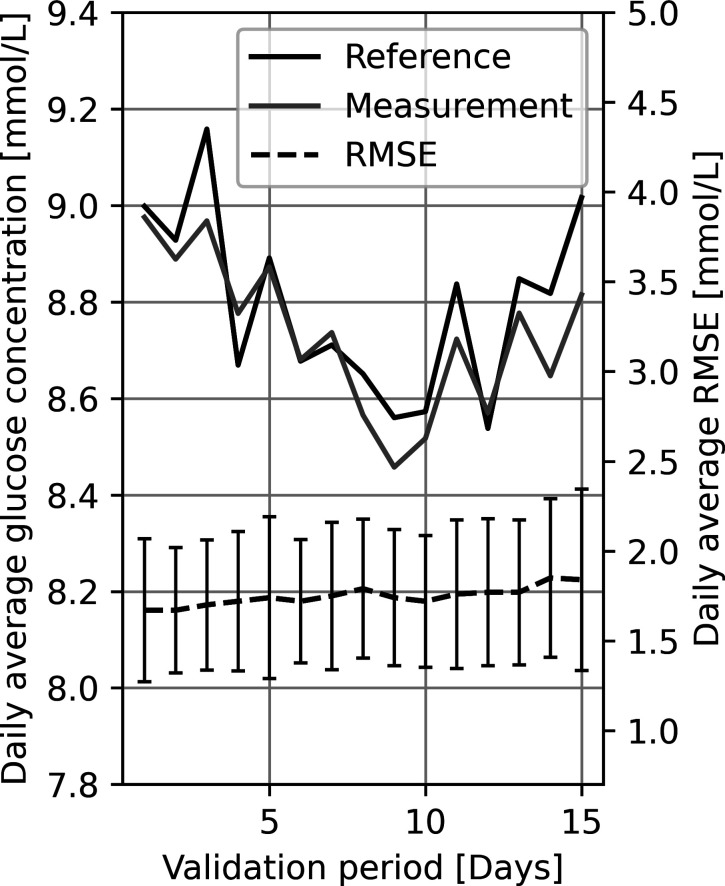
Calibration stability. Comparison between the daily mean of the measured and reference glucose value and the subject-wise, average RMSE for the 160 subjects for a validation period of 15 days. The bars on the RMSE curve represent the standard deviation.

For a sensor to be considered applicable for practical non-invasive glucose monitoring, it is necessary to show calibration stability. Thus, accuracy should not depend on frequent recalibration but remain stable over days and weeks. In the present work, we have achieved measurement stability over a period of 15 days after finalized calibration. Figure 2 shows the time course of the daily root-mean-squared error (RMSE), mean glucose measurement, and reference value in the validation phase of 15 days for all subjects at home or work, without close professional supervision. The measurement values are seen to closely follow the reference values within 0.2 mmol/L. In the entire 15 day validation period, the measurement accuracy remained stable, with only a slight increase in RMSE from 1.68 to 1.84 mmol/L, thus corresponding to a reduction in measurement accuracy of 9.5%.

### Performance in Subjects with Type 1 and Type 2 Diabetes

**Figure 3:**
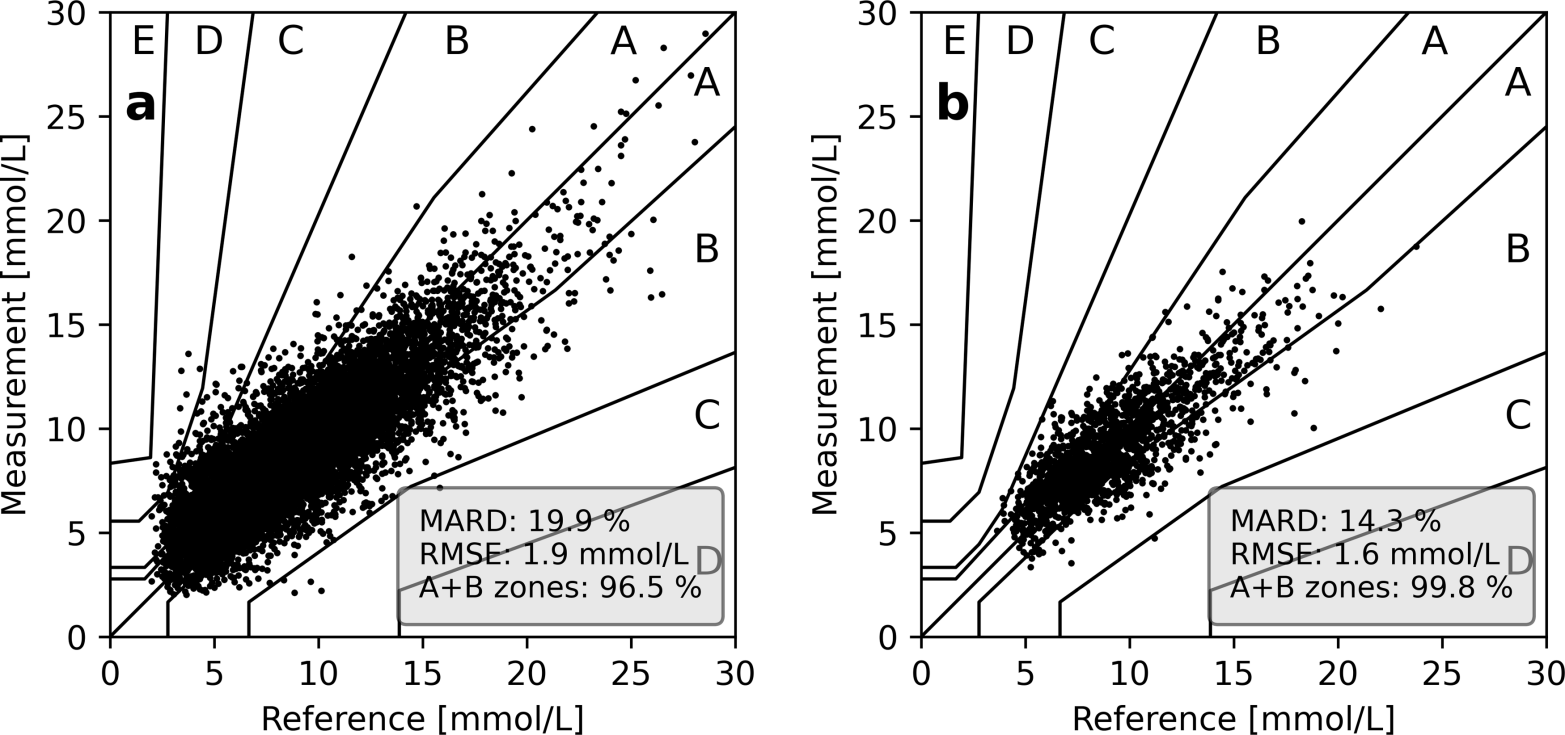
Measured glucose concentrations plotted as a function of reference values in a consensus error grid for all type 1 (a) and type 2 subjects (b). The reference glucose value is obtained as the average of two blood glucose measurements (Contour Next One, Ascensia), whereas the corresponding glucose measurement is the result of the PLS regression model applied to three pre-processed NIGM spectra.

The clinical study involved 160 subjects, 137 with type 1 on intensive insulin therapy or insulin pumps and 23 with type 2 diabetes on insulin or antidiabetic medication. For the first group with type 1 diabetes, the overall accuracy of measurements is given in the consensus error plot of Figure 3a, where 96.5% of the points fall into zones A + B, while the typical indices of accuracy, the mean absolute relative difference (MARD) and RMSE, over the 15 days were 19.9% and 1.9 mmol/L, respectively. For the cohort of type 2 diabetes subjects, NIGM measurements showed points within A + B in a consensus error grid, MARD, and RMSE of 99.8%, 14.3%, and 1.6 mmol/L, respectively. As shown in Table 1, RMSE and MARD were strongly dependent on the range of the glucose concentration. This is particularly emphasized by the MARD for the group of subjects with type 1 diabetes on intensive insulin therapy with the glucose values below 3.9 mmol/L (i.e., hypoglycemia). However, this is a feature of the MARD metric accentuating the performance in the lower glucose ranges.^14^ The total collective of 160 subjects of the study (all types of diabetes and forms of therapy) was grouped in age ranges, gender, and skin colors according to the scale of Fitzpatrick. As Table 2 shows, there are no major changes in the indices of performance for these parameters. In view of the limited numbers, these data need further confirmation.

**Table 1:** Performance of the Non-Invasive Glucose Sensor for Three Glucose Reference Intervals, Corresponding to Hypo-, Eu-, and Hyperglycemic Ranges^a^

group	interval [mmol/L]	points [number]	RMSE [mmol/L]	MARD [%]
overall	all points	12,374	1.9	19.1
	0–3.9	542	2.5	58.8
	4.0–10.0	8141	1.6	18.9
	10.1–30.0	3691	2.3	13.9
type 1	all points	10,612	1.9	19.9
	0–3.9	537	2.5	58.9
	4.0–10.0	6897	1.7	19.6
	10.1–30.0	3178	2.4	14.1
type 2	all points	1762	1.6	14.3
	0–3.9	5	2.1	51.8
	4.0–10.0	1244	1.4	15.0
	10.1–30.0	513	2.1	12.4

a The results are shown for all 160 subjects and when divided into the type 1 and 2 segments.

**Table 2:** Performance of the Non-Invasive Glucose Sensor as a Function of Age, Gender, and Skin Color for All 160 Subjects^a^

group	subjects [number]	RMSE [mmol/L]	MARD [%]	A + B [%]
all	160	1.9	19.1	97.0
T1 with pump	69	2.0	19.9	96.6
T1 without pump	68	1.9	19.9	96.4
T2	23	1.6	14.3	99.8
age: 18–31	29	2.0	19.2	96.7
age: 32–46	51	1.9	19.3	97.1
age: 47–61	51	1.9	19.4	96.8
age: 62–76	29	1.8	18.3	97.4
male	72	1.9	19.2	96.9
female	88	1.9	19.1	97.1
skin: I	3	1.6	18.8	98.0
skin: II	48	2.0	18.4	97.9
skin: III	88	1.9	19.5	96.5
skin: IV	19	1.8	19.3	96.9
skin: V	2	1.9	17.9	97.0

a T1 and T2 mark people with type 1 and type 2 diabetes, respectively. Age groups are in years of age. Skins I through V denote the skin color from the lightest to the darkest skin complexions, respectively, according to the Fitzpatrick scale. The A + B column represents the percentage of the representative number of points in the A and B zones in a consensus error grid.

### Individual Performance

**Figure 4:**
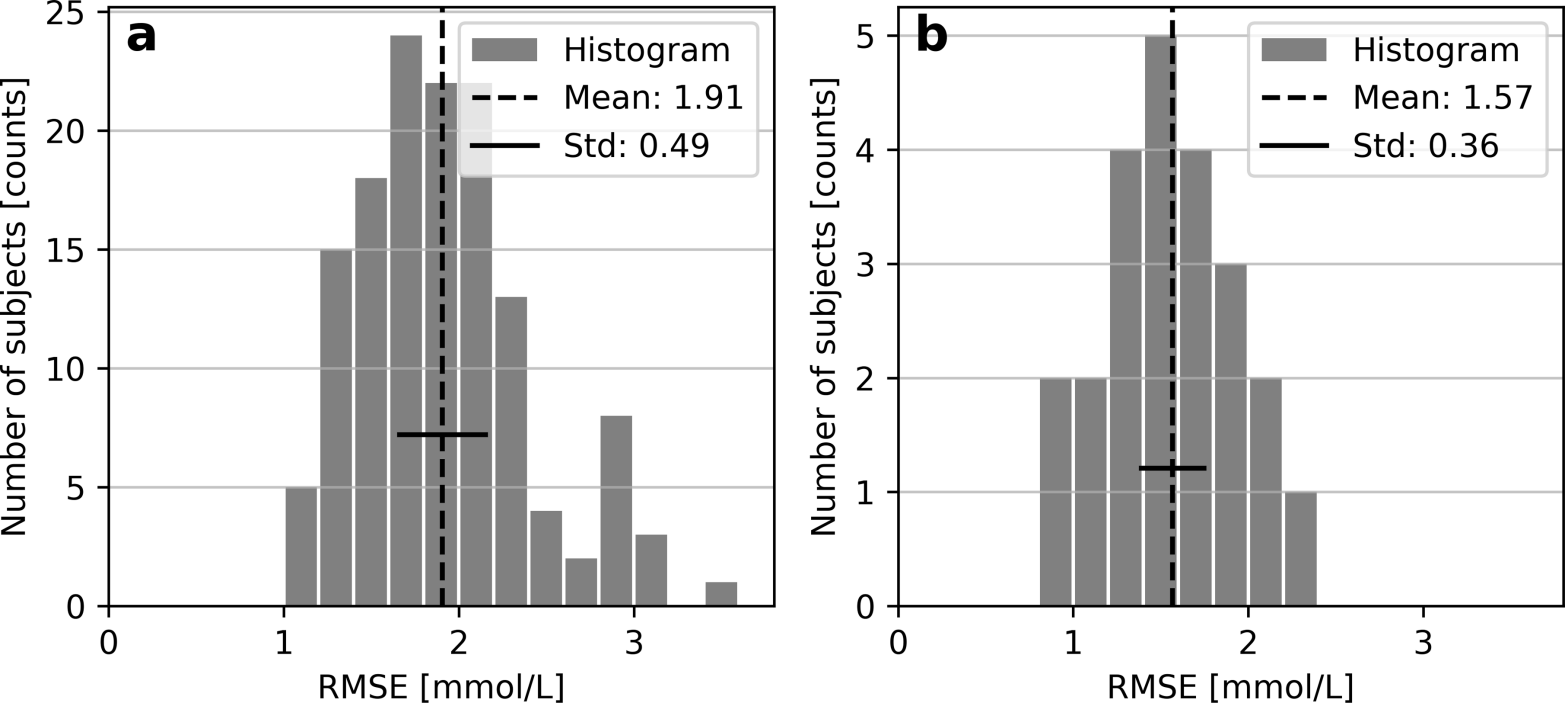
Histograms of subject-wise RMSE values for (a) 137 subjects with type 1 diabetes and (b) 23 subjects with type 2 diabetes.

The above results describe the performance of pooled data, acquired by uniting measurements from enrolled subjects. To assess the homogeneity of performance in the two subject collectives, histograms were established for subject-wise RMSE, as shown in Figure 4. Noticeable variation exists in RMSE, where subjects with type 1 diabetes feature an RMSE of 1.9 ± 0.5 mmol/L (mean ± standard deviation). The subjects with type 2 diabetes show a slightly more consistent performance, with an RMSE of 1.6 ± 0.4 mmol/L. It should be noted that with the available metadata at hand (such as gender, age, and skin color; see Table 2), we have only been able to establish a clear relation between performance and the type of diabetes. The intra-group performance variations are a result of many influential parameters, where particularly the subject-specific glucose dynamics and biological properties are recognized as some of the key factors. For example, the thickness of the outer most skin layer, the dead stratum corneum layer, is 166 ± 40 μm on thenar,^9^ meaning that the Raman spectra from different subjects feature different proportions of the signal from the dead and living parts of the skin, which influences the raw signal-to-noise ratio. We note that the inter-subject variation of stratum corneum contribution to the Raman signal can, in principle, be mitigated by adjusting the confocal collection depth for each sensor to the specific subject. However, it was the purpose of this study to test one nominal sensor configuration (collection depth of 280 μm) to be used by all. As another example of biological variation, skin autofluorescence is a noticeable contributor to the in vivo Raman spectra, and as the fluorescence level is subject-dependent (see Figure 1c), it contributes to the shot-noise in different ways from subject to subject. To complicate matters, the fluorescence level is also subject to photobleaching during a measurement, and this fluorescence decay is seen to vary both within and between subjects (for illustrative examples, see Figure S1).

### Regression Vector of the Calibration Model

**Figure 5:**
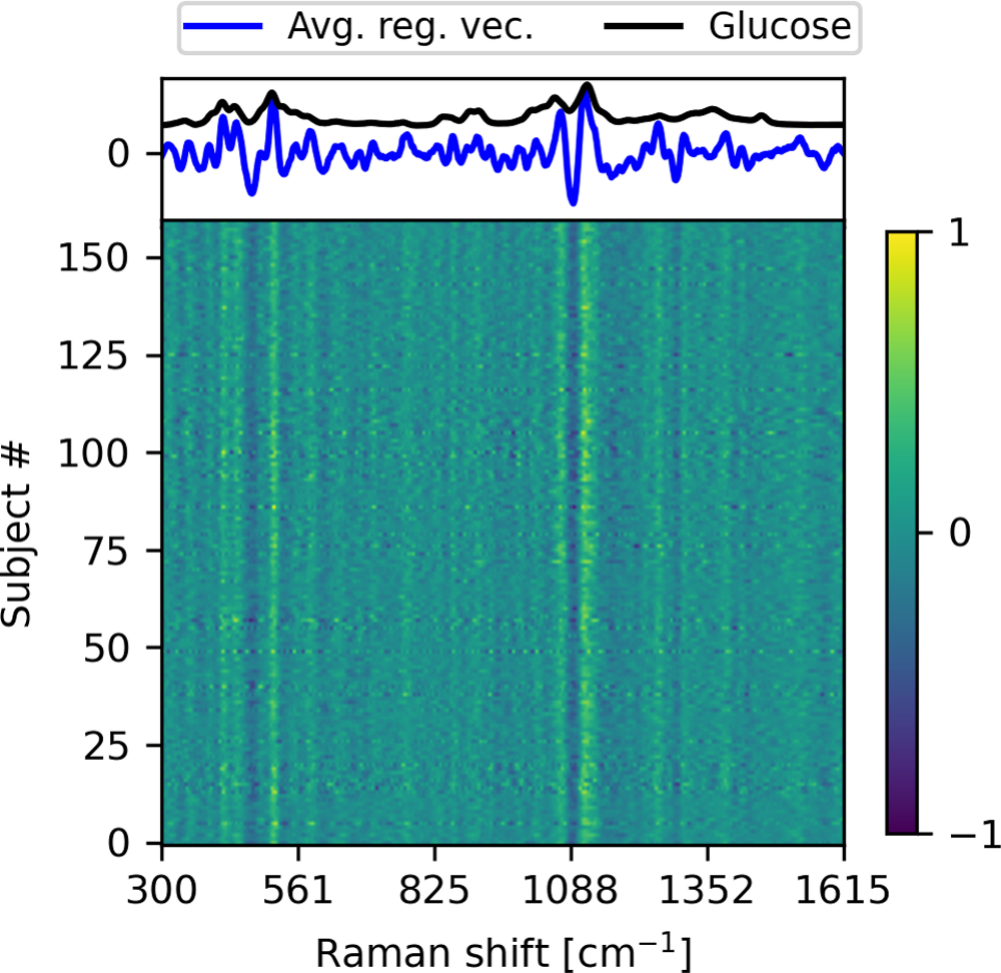
Regression vectors from the individual prediction models. The top shows the Raman spectrum for glucose and the average regression vector obtained from the PLS prediction models. The regression vector is seen to mimic the significant peaks in the glucose spectrum. This is consistent for all 160 subjects as demonstrated on the color-coded map.

By controlling all external factors that may influence a Raman spectrum, such as temperature, skin inhomogeneity, and body movement, it has previously been shown that the glucose fingerprint though weak is directly visible in in vivo skin spectra.^2^ In this study, the influence of a multitude of external perturbations precludes the possibility to directly view the change in the skin Raman spectrum as implied by a change in the glucose concentration. Instead, insight into the relation between spectral and glucose changes can be sought via interpretable multivariate regression techniques. In our case, we based the subject-wise calibration models on partial least squares (PLS) regression in which the associated regression vector represents the importance of the different regions of the Raman spectrum when correlating with glucose reference values. Figure 5 shows the regression vectors of the 160 PLS models (one for each subject), which convincingly demonstrates that despite noticeable inter-subject spectral variation, the PLS algorithm shows a consistent regression vector, thus underlining that the spectrum-glucose correlation is not spurious but a consequence of the glucose fingerprint present in all measured skin spectra. Furthermore, by comparing the subject-averaged regression vector with a Raman spectrum of a glucose solution, it is evident that the most influential spectral areas (i.e., where the regression vector has the largest absolute values) coincide with the main Raman peaks of glucose. In this regard, it is important to recognize that the regression vector should not simply mirror the glucose spectrum as the complex matrix of the skin requires the regression vector to account for non-glucose spectral features and variations.

It is worth noting that despite the similarity between the individual regression vectors, the underlying PLS regression models do not necessarily feature the same number of latent variables. For the 160 subjects, the distribution of the number of latent variables is 20.8 ± 2.7, while subgroup analysis regarding diabetes type shows 20.6 ± 2.8 and 21.7 ± 2.0 for type 1 and 2, respectively. In all cases, the number of latent variables is high, which is a signature of the complexity of the problem with a small glucose signal residing in a largely varying thenar Raman spectrum.

## Discussion

Discomfort and burden from multiple daily skin punctures have for years been a strong motivation to develop technologies for non-invasive glucose determination.^15^ While the minimal-invasive enzyme electrode, after about 40 years of research and development, found its way into practical routine,^16−19^ non-invasive glucose testing, despite the many different, technically sophisticated approaches,^20^ has not yet progressed to widespread practical use.

Here, we present a portable instrument manufactured in series that gives a satisfactory performance in the hands of subjects with type 1 and type 2 diabetes. Its accuracy, as demonstrated on consensus error grids and by MARD and RMSE values, is comparable to what was found in early continuous glucose monitoring studies with enzyme electrodes, where MARD values between 8.8 and 19.9% have been reported in home use.^14^ One must also take into account that in continuous monitoring, glucose kinetics are subject to mathematical correction to counteract the time lag associated with glucose transfer from capillary to interstitial space.^11^ Contemporary CGMs employ trend information to correct for the time delay.^11^ This type of correction is not possible with the current intermittent measurements of NIGM. However, when NIGM is operated in a semi-continuous mode, there is no principal hindrance to improve accuracy by time-series analysis and consideration of glucose dynamics.^21^ In fact, an observed accuracy difference of ∼0.3 mmol/L between groups with type 1 and type 2 diabetes (see Figure 3) is mainly ascribed to the former group experiencing larger and steeper glucose fluctuations, which could be alleviated by such corrective means.

As observed in CGM, MARD as the index for accuracy is influenced by the range as well as by rapid changes of the glucose concentration.^14^ Our data suggest similar effects in NIGM. The group of subjects with type 1 diabetes was a considerable part of the glucose measurements in the hypoglycemic range below 3.9 mmol/L, contributing to a relatively high MARD value of 19.9%. In contrast, the 23 subjects with type 2 diabetes showed a smaller glucose spread and had only 0.12% of points under 3.9 mmol/L. The encouraging performance metrics of 99.8% of points in zones A + B on the consensus error plot of Figure 3b, an RMSE of 1.6 mmol/L, and MARD of 14.3% suggest the qualification of the instrument for use in type 2 diabetes.

The obtained accuracies for the subjects with type 1 and type 2 diabetes are in the upper and the middle of the MARD range as previously reported for CGM with enzyme electrodes in home use.^14^

The presented performance metrics represent average values over the entire glucose range of reference values (∼2 to 30 mmol/L). It is important to clarify that individual PLS models are built on data available from the calibration days, meaning that the subject-specific distribution of glucose reference values dictates how the models emphasize different glucose intervals. Thus, the best measurement accuracy is achieved for glucose values around 8 mmol/L, which coincides with the glucose value most frequently occurring in the calibration data (see Table 1 and Figure S2). Though not sought in this work, we note that the dependence of accuracy on the glucose value can be eliminated by constructing regression models on a controlled distribution of reference values.^22^

The presented NIGM sensor technology is based on a confocal Raman setup that converts recorded thenar spectra to quantitative glucose values through the use of chemometrics. This approach is fundamentally different from commercial, home-use (invasive) glucose monitors that are based on enzymatic electrochemical technology, where a generated electrical current is proportional to the surrounding glucose level.^23^ This is also the basic working principle of novel wearable sweat glucose sensors.^24,25^ The electrochemical technology is therefore based on univariate regression, meaning that any spurious chemical activity, adding or subtracting from the primary process of chemical conversion of glucose by the enzyme, biases the glucose measurement. As such, the issue of interferences must be combated on the hardware level, which is typically achieved by using an enzyme that is highly sensitive to glucose and by coating the electrode with a permselective membrane.^23^ The Raman + chemometrics approach is altogether different as no single point in the spectrum determines the glucose value, but a multitude of signals contributes to the determination of a single glucose value. The sensitivity toward glucose is not inherently present in the thenar spectra, but the specificity is achieved through training of the multivariate regression model. By feeding the model with a multitude of paired spectra and reference glucose values acquired over many days, the influences from natural biological variation and environmental conditions are separated from the glucose variability, hence creating robust models that are sensitive to glucose. The robustness and glucose sensitivity, as demonstrated by the 15 day calibration stability and accurate glucose measurements, are thus achieved through mathematical means.

The 15 day stability of calibration, the consistent spectrum-glucose correlation, and the lack of major effects of age, gender, and skin color on performance, as shown in Table 2, unequivocally demonstrate that the combination of Raman spectroscopy and chemometrics can be configured for practical use. The presented results are based on PLS models that are built on 26 days of calibration data. The extended calibration period originates from the study setup, featuring six measurements per day, and the requirement of a certain amount of data to ensure predictive power and calibration stability of the subject-wise PLS models.^26^ That said, it is important to emphasize that the PLS models are not crucially sensitive to the 26 days of calibration. For example, the number of calibration days (and the size of the calibration set accordingly) can be reduced to 20 or 14 days with a respective increase in the average, subject-wise RMSE over the 15 day validation period of 2.4 and 10.7% (for further details, see Figure S3). It is expected that the calibration requirement can be significantly reduced by utilizing calibration transfer techniques and/or creating robust regression models by combining data from multiple subjects and devices.^27^

## Conclusions

We have shown that Raman spectroscopy, coupled with multivariate data analysis, is well suited for home-use non-invasive glucose monitoring in people with diabetes. We developed a robust Raman-based, portable sensor for intermittent glucose determination that has proven to be successful in the hands of lay people, irrespective of age, gender, and skin color. Crucially, the sensor technology can be calibrated for real-life usage, which in this work is demonstrated by a measurement accuracy that remains stable over a 15 day validation period. The glucose sensor is still in development, and our focus is on further miniaturization, further improvement of accuracy, extended calibration stability, and a reduced calibration scheme. As a final remark in relation to the ever-present discussion of the beginning of the non-invasive era in diabetes management, it is interesting to note that the presented results convincingly corroborate with a recent review that foresees Raman spectroscopy to be the most promising technology for non-invasive glucose monitoring.^28^

## Materials and Methods

### Instrumentation

The spectral acquisition was performed using a custom-built confocal Raman setup of external dimensions of 168 mm (*l*) × 130 mm (*w*) × 62 mm (*h*). The optical module, as depicted in Figure 1b, consists of a spectrometer and a probe assembled into one unit (Wasatch Photonics, USA). The thenar of the hand was placed on a 500 μm-thick magnesium fluoride window for the measurements. The output from the continuous-wave diode laser (Beijing RealLight Technology, China), emitting light at a wavelength of 830 nm with a power of 300 mW, was first collimated, and unwanted spectral side lobes and fluorescence were removed by a clean-up filter. The laser light was then transmitted by the dichroic mirror and finally focused (by the *f*/0.55 lens) just below the skin surface. Meanwhile, both the intense reflected/scattered light, fluorescence, and generated Raman photons were collected by the *f*/0.55 lens; the dichroic mirror and the long-pass filter ensure that only the latter two contributions to the spectrum were focused by the lens on the entrance slit of the spectrometer that also functions as the pinhole in our confocal setup. The spectrometer has an *f*-number of 1.3 with a spectral resolution better than 1 nm in the measurement range of ∼850 to 960 nm. Finally, the dispersed light was recorded by a CCD image sensor (Hamamatsu, Japan) that was temperature-stabilized at 20 °C.

### Participants

The clinical study was performed at Institute for Diabetes Technology at University of Ulm, Germany, Steno Diabetes Center Copenhagen and Steno Diabetes Center Odense, Denmark according to the Declaration of Helsinki and the Guidelines for Good Clinical Practice. One hundred sixty consecutive persons with manifested type 1 and type 2 were recruited and asked for written consent. Exclusion criteria were severe hypoglycemia in the past 3 months; hypoglycemia unawareness; severe diabetes-related complications (e.g., advanced autonomic neuropathy, kidney disease, foot ulcers, legal blindness, or symptomatic cardiovascular disease as evidenced by a history of cardiovascular episode(s)); systemic or topical administration of glucocorticoids for the past 7 days; pregnancy or lactation period; known severe allergy to medical-grade adhesive or isopropyl alcohol (used to clean the skin); inability to comply with the study procedures (due to, e.g., psychiatric diagnoses, lack of cognitive ability, alcohol dependency, drug use, or psychosocial overload); inability to hold the arm or hand still (including tremors and Parkinson’s disease); and extensive skin changes, tattoos, or diseases on the right thenar. All subjects were screened with a skin tone sensor (DEESS Demi II GP531, Shenzen GSD Tech Co., Ltd., China) for skin color I to V according to the Fitzpatrick scale.^29^ One hundred thirty-seven persons with type 1 diabetes, used to intensive treatment with blood glucose self-monitoring 4–6 times per day, rapid mealtime insulin, and long-acting insulin at bedtime or pump use, were instructed in the use of the device. The cohort of 23 subjects with type 2 diabetes on oral antidiabetic drugs and/or insulin was under a similar test regimen as the group with type 1 diabetes.

### Ethical Standards

The study was approved by the local ethical committees, the German Federal Institute for Drugs and Medical Devices, and the Danish Medicines Agency. It was registered as no. 2020040420 (DK) and with EUDAMED no. CIV-20-04-032405.

### Study Design

The study period was 41 days, where the first 26 days of the study were used for calibration, while the remaining 15 days were used for validation. On each day, subjects performed six measurement units, each comprising two reference capillary tests and three NIGM scans in the sequence BGM reference, NIGM, NIGM, BGM reference, and last, NIGM. The NIGM scans lasted for 75 s each, but the measurement time can easily be reduced without noticeably affecting measurement performance (see Figure S4). All subjects remained unaware of the NIGM readings. After instruction on the use of the NIGM device, there was no further professional supervision during the sessions for the days at home or work. Capillary glucose, as standard for calibration and parallel measurement with NIGM, was measured with the Contour Next One system (Ascensia, Switzerland). Accuracy in the hands of subjects was found to correspond to a MARD of 5.6%.^30^ Control solution measurements were performed on the test strips for every new strip vial opened before handing test strips to subjects. The raw data were transmitted to RSP Systems, Odense, Denmark for further evaluation.

### Data Analysis

The relationship between recorded Raman spectra and associated BGM references was established through PLS regression.^31^ The data analysis was centralized in Python using the scikit-learn package. A single NIGM scan involved a series of recorded Raman spectra, while a single measurement unit comprised three NIGM scans. The study comprised 41 measurement days, where each day encompassed six measurement units. Thus, the starting point of the data analysis was a large database of thenar Raman spectra that initially underwent cleaning/filtering. The cleaning step involved removal of saturated spectra, spike removal, and deletion of NIGM units in which the difference in the two BGM reference values was above 1.5 mmol/L. The latter represented an unusual high variation in consecutive BGM references and, for this reason, was treated as error-prone reference values. After the initial cleaning, the spectra of each scan were averaged to a single spectrum, normalized to unit Euclidean norm, and aligned to a Raman axis of 300–1615 cm^–1^ in 700 equidistant points (i.e., spectral features). The spectra were further processed by Savitzky–Golay smoothing (five-point window, first-order polynomial) and corrected for varying fluorescence backgrounds by second-order extended multiplicative scatter correction (EMSC).^32^ The BGM reference of a measurement unit was found by simple averaging of the two reference values.

To improve model construction and prediction, the dataset was analyzed for the presence of outliers. Spectral outliers were identified by calculation of the Q-residuals and Hotelling’s T2s and subsequently compared to the 99% confidence intervals.^33^ If more than one scan of a measurement unit was identified as an outlier, then the whole unit was removed. As the final preprocessing step, the spectra and reference values were mean-centered. The PLS regression model was trained on the preprocessed scan spectra, where the three spectra of a measurement unit refer to the same reference value. The number of PLS components was determined from minimization of the root-mean-squared error (RMSE) of 20-fold, contiguous cross-validation. During validation of the calibration model, the prediction of a measurement unit was obtained by averaging the underlying scan predictions, as obtained by entering the scan spectra into the PLS model. It is important to recognize that the dataset consists of 26 and 15 days of calibration and validation data, respectively, that were kept separate during the data analysis. For example, the extended multiplicative scatter correction reference, outlier model, mean-center reference, and PLS regression model were all based on calibration data, while the validation data was solely used for independent validation of the predictive performance.

## Data Availability

All data needed to evaluate the conclusions in the paper are present in the paper and/or the Supporting Information. Additional data related to this paper may be requested by contacting the corresponding author.

## Supporting Information Available

Illustration of fluorescence decay during measurements, measurement accuracy vs glucose reference intervals, performance vs calibration days, and performance vs measurement time (PDF)
