# Supplementary material for: Accurate Post-Calibration Predictions for Noninvasive Glucose Measurements in People Using Confocal Raman Spectroscopy
Source: ACS Sens. 2023 Mar 6;8(3):1272–9. doi: 10.1021/acssensors.2c02756 (PMC10043934; doi:10.1021/acssensors.2c02756)
Supplement: Supplementary file 1 — se2c02756_si_001.pdf [file se2c02756_si_001.pdf]

# Supporting Information

# Accurate post-calibration predictions for noninvasive glucose measurements in people using confocal Raman spectroscopy

Anders Pors[†#], Kaspar G. Rasmussen[†#], Rune Inglev[†], Nina Jendrike[§], Amalie Philipps[†], Ajenthen G. Ranjan[‖], Vibe Vestergaard[⊥], Jan E. Henriksen[⊥], Kirsten Nørgaard[‖], Guido Freckmann[§], Karl D. Hepp[ǂ], Michael C. Gerstenberg[†], and Anders Weber[†*]

[†]RSP Systems, Sivlandvænget 27C, 5260 Odense, Denmark

[§] Institute for Diabetes Technology at University of Ulm, Lise-Meitner-Straße 8/2, 89081 Ulm, Germany

[‖]Steno Diabetes Center Copenhagen, Borgmester Ib Juuls Vej 83, 2730 Herlev, Denmark

[⊥]Steno Diabetes Center Odense, Kløvervænget 10, 5000 Odense, Denmark

[ǂ]University of Munich (emeritus), Geschwister-Scholl-Platz 1, 80539 Munich, Germany

#These authors contributed equally.
*Corresponding author: andersw@rspsystems.com

**This PDF file includes:**

Fig. S1. Illustration of fluorescence decay during measurements.

Fig. S2. Measurement accuracy vs glucose reference intervals.

Fig. S3. Performance vs calibration days.

Fig. S4. Performance vs measurement time.

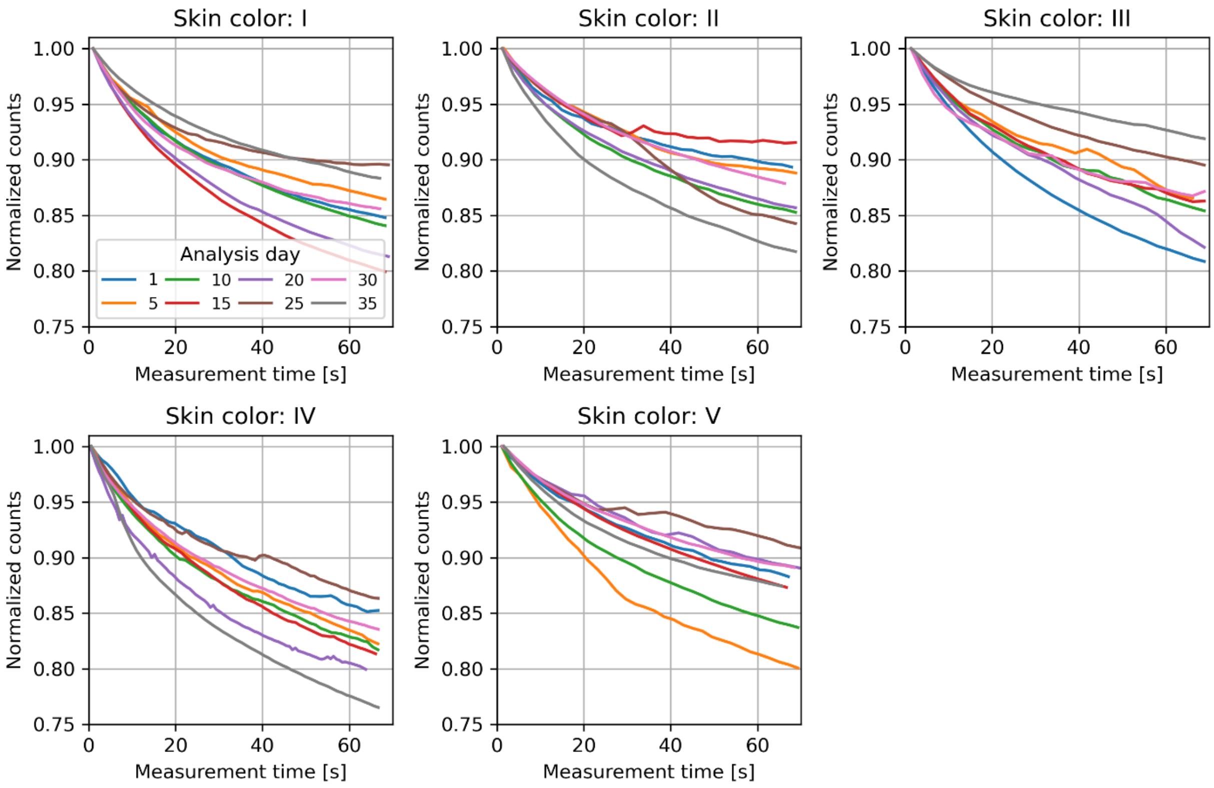


**Figure S1.** Illustration of fluorescence decay during measurements. The figures exemplify how the Raman signal decreases during a measurement scan for five subjects with different skin color (the same subjects as shown in Figure 1c). The signal decay owes to quenching of the fluorescence signal during the measurement. The signal decay is not systematically evolving during the study days but is a factor that varies both within and between subjects.

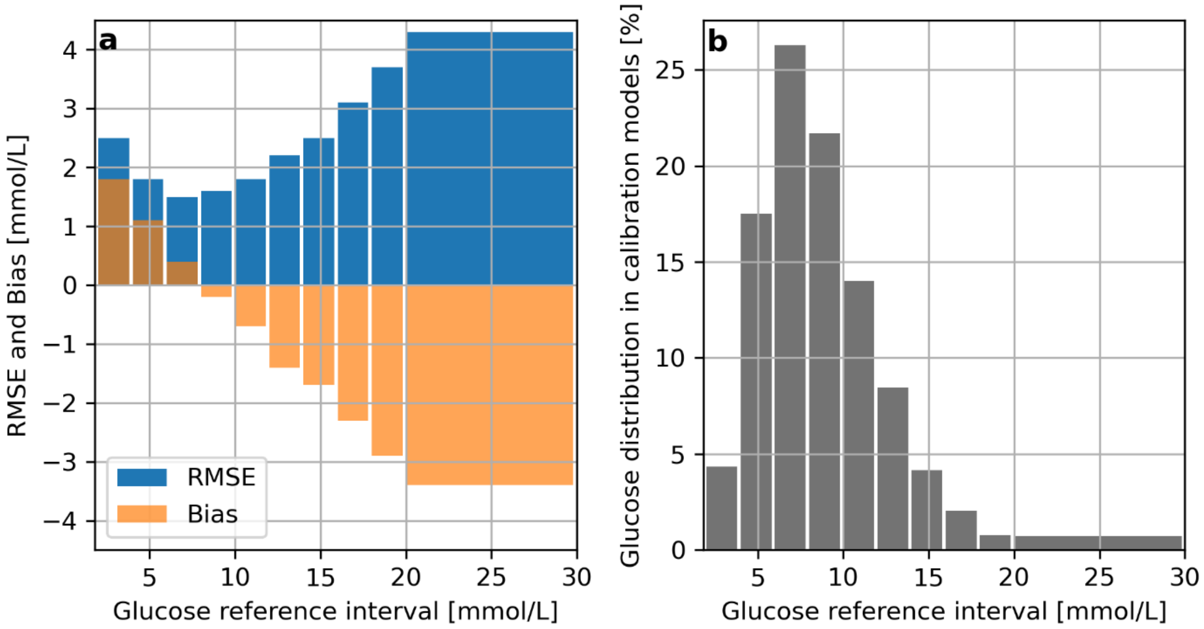


**Figure S2.** Measurement accuracy vs glucose reference intervals. (a) The root mean squared error (RMSE) and bias for 15 days pooled validation data from 160 subjects when reference concentrations are split into intervals of 2mmol/L for reference values below 20mmol/L and one large interval above. It is seen that measurement accuracy is noticeably dependent on the reference values, with best RMSE of 1.5mmol/L around reference values of 8mmol/L. As the reference values move away from 8mmol/L, an increasing part of RMSE owes to a bias that is positive (negative) for low (high) glucose values, hence indicating a general overestimation (underestimation) of the glucose value. (b) The glucose reference distribution of pooled calibration data. It is seen that interval-based RMSE in validation reflects the distribution of reference points in calibration, where intervals with most data points show the best RMSE.

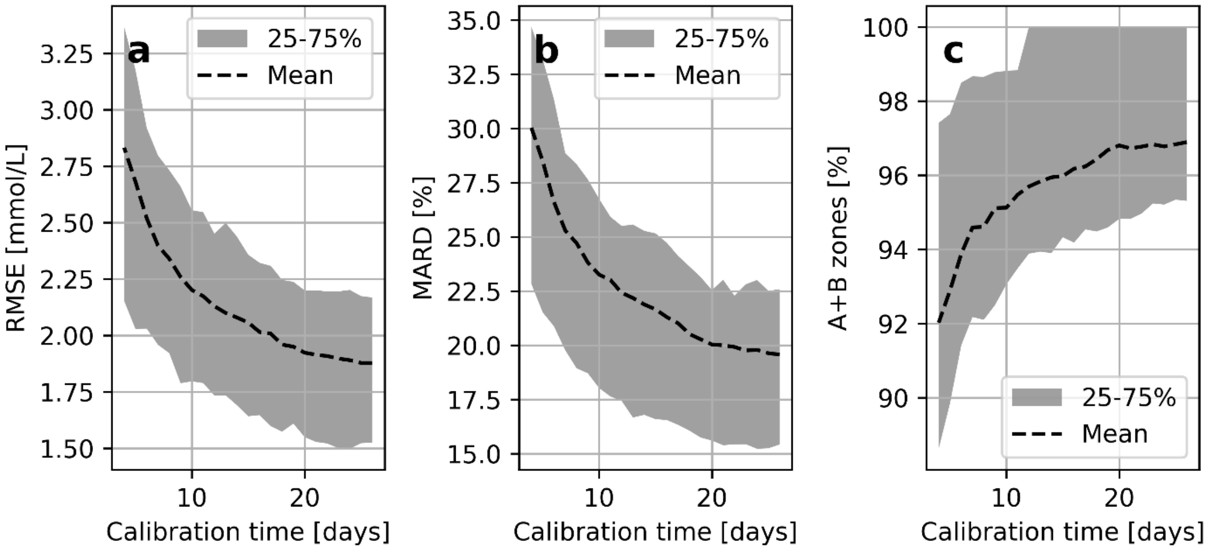


**Figure S3.** Performance vs calibration days. The dashed lines represent the average, subject-wise (a) RMSE, (b) MARD, and (c) percentage in A+B zones over a fixed 15-day validation period when the number of calibration days is varied between 4 and 26 days. The calculations include 160 subjects, where the gray band displays the 25-75% quantile range of subject-wise performance metrics. With nominally six measurement units per calibration day, it is important to note that a reduction in number of calibration days correspondingly reduces the size of the calibration set.

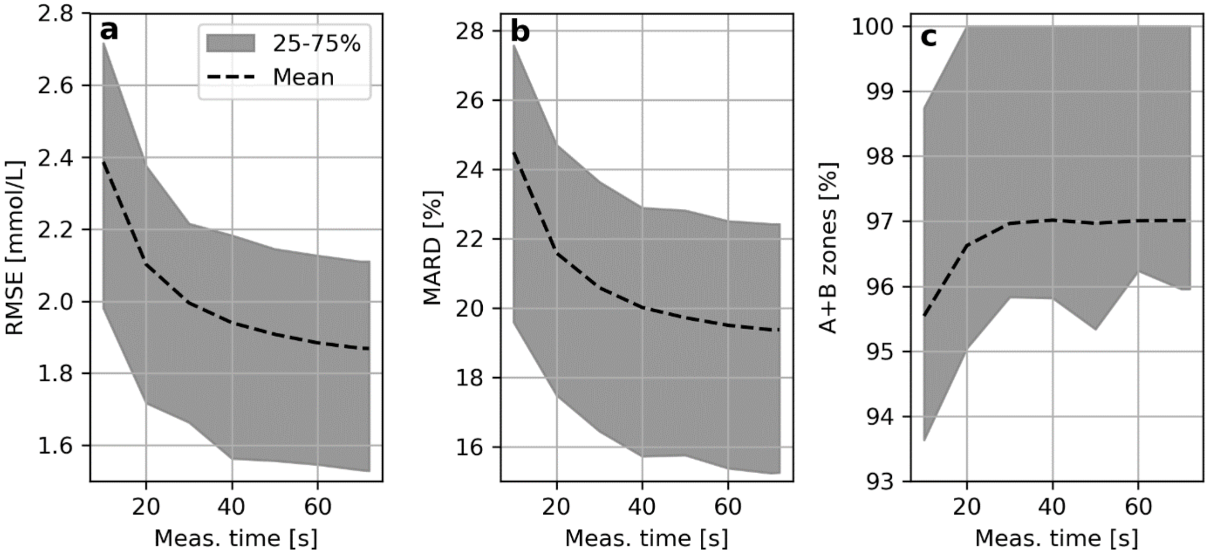


**Figure S4.** Performance vs measurement time. The dashed lines represent the average, subject-wise (a) RMSE, (b) MARD, and (c) percentage in A+B zones over a fixed 15-day validation period when the measurement time per scan is varied. The calculations include 160 subjects, where the gray band displays the 25-75% quantile range of subject-wise performance metrics.
